# A comparison of hepato-cellular *in vitro* platforms to study CYP3A4 induction

**DOI:** 10.1371/journal.pone.0229106

**Published:** 2020-02-27

**Authors:** Beyza Bulutoglu, Camilo Rey-Bedón, Safak Mert, Lipeng Tian, Yoon-Young Jang, Martin L. Yarmush, O. Berk Usta

**Affiliations:** 1 Center for Engineering in Medicine, Massachusetts General Hospital, Harvard Medical School and Shriners Hospitals for Children, Boston, Massachusetts, United States of America; 2 Department of Oncology, The Sidney Kimmel Comprehensive Cancer Center, Institute for Cell Engineering, School of Medicine, Johns Hopkins University, Baltimore, Maryland, United States of America; 3 Department of Biomedical Engineering, Rutgers University, Piscataway, New Jersey, United States of America; University of Tampere, FINLAND

## Abstract

*In vitro* studies of drug toxicity and drug-drug interactions are crucial for drug development efforts. Currently, the utilization of primary human hepatocytes (PHHs) is the *de facto* standard for this purpose, due to their functional xenobiotic response and drug metabolizing CYP450 enzyme metabolism. However, PHHs are scarce, expensive, require laborious maintenance, and exhibit lot-to-lot heterogeneity. Alternative human *in vitro* platforms include hepatic cell lines, which are easy to access and maintain, and induced pluripotent stem cell (iPSC) derived hepatocytes. In this study, we provide a direct comparison of drug induced CYP3A4 and PXR expression levels of PHHs, hepatic cell lines Huh7 and HepG2, and iPSC derived hepatocyte like cells. Confluently cultured Huh7s exhibited an improved CYP3A4 expression and were inducible by up to 4.9-fold, and hepatocytes differentiated from human iPSCs displayed a 3.3-fold CYP3A4 induction. In addition, an increase in PXR expression levels was observed in both hepatic cell lines and iPSC derived hepatocytes upon rifampicin treatment, whereas a reproducible increase in PXR expression was not achieved in PHHs. Our results indicate that both hepatoma originated cell lines and iPSCs may provide alternative sources to primary hepatocytes, providing reliable and reproducible results for CYP3A4/PXR metabolism, upon *in vitro* maturation. This study may serve as a guide for the selection of suitable and feasible *in vitro* platforms for drug-drug interaction and toxicology studies.

## Introduction

Cytochrome P450 (CYP450) family comprises 57 genes in the human genome which produce vital anabolic and catabolic enzymes, especially in the liver. Of the 18 protein families of CYP450, CYP1, 2 and 3 are involved in the breakdown of more than 80% of all prescribed drugs [1]. Particularly, CYP3A4 is involved in the breakdown of more than 50% of clinically approved medications [2, 3]. A wide range of xenobiotics are able to induce the transcription of CYP3A4 through the pregnane X receptor (PXR; also known as NR1I2), a member of the nuclear receptor protein family [4]. When a xenobiotic contacts the ligand-binding pocket of PXR, this complex is translocated into the nucleus where it binds to a response element on the CYP3A4 promoter as a heterodimer with the 9-cis retinoic acid receptor (RXR) [5]. When multiple drugs are present, the upregulation of the CYP3A4 enzyme by one drug can affect the metabolism of the other co-administered compound resulting in a drug-drug interaction (DDI). For instance, when PXR induces the expression of CYP3A4, this can cause the accumulation of toxic intermediate metabolites of CYP3A4 metabolizing drugs, resulting in hepatic toxicity [6]. On the other hand, increased metabolism of certain drugs—through elevated levels of CYP3A4—can severely reduce their efficacy. Accordingly, determining if a drug is a PXR activator is critical in drug discovery, as DDIs are one of the main causes for drug withdrawals [7].

Primarily, hepatic *in vitro* models are used to determine potential DDIs in the liver by assessing enzyme gene expression after drug exposure. When a drug is predicted to cause severe DDIs, multiple assays are performed to identify and minimize this characteristic without diminishing the efficacy of the drug [8, 9]. *In vitro* models usually employ primary human hepatocytes (PHHs), hepatic cell lines, and stem cell derived hepatocytes. PHHs remain the gold standard for assessing CYP3A4 activity and regulation, as they retain a significant portion of their *in vivo* CYP450 metabolism after the isolation procedure [10]. Nevertheless, isolated PHHs present high donor to donor variability, are scarce and expensive due to their inability to proliferate *in vitro*, and lose their phenotype after a few days of culturing in a regular 2D monolayer [11]. In the past decades, there have been multiple approaches to culture hepatocytes in 2.5D and 3D structures, which have helped extend the hepatic phenotype of PHHs to over 1 to 4 weeks [12–15]. This allows for the study of long-term drug exposure and complex disease models *in vitro* [12]. Additionally, cryopreservation has been optimized to offer a systematic and continuous supply of PHHs. However, cryopreserved PHHs can lose their plateability after the cryopreservation process and lose ~50% of their CYP450 activity each day after thawing, stabilizing at 10–20% [11, 16]. Moreover, their availability is dependent on organ donation, which is scarce, and the isolation is performed only on marginal livers rejected for transplantation [11, 16].

To overcome some of these difficulties and complications, human hepatoma cell lines have been developed and characterized to provide a stable and unlimited source of hepatocyte like cells. HepG2 is the most widely used cell line in pharmaco-toxicological research. It originated from the differentiated hepatocellular carcinoma of an American 15-year-old Caucasian patient. This cell line possesses many hepatic functions, including synthesis and secretion of plasma proteins, bile acid synthesis, and insulin signaling [17–19]. Nevertheless, the CYP450 enzyme expression of HepG2 cells is significantly low in comparison to PHHs. Particularly, their basal CYP3A4 levels are lower than PHHs, but they have been shown to be induced by up to 4-fold when treated with rifampicin [19–21]. This has led HepG2 to be used in drug response and PXR regulatory mechanism studies [21–23].

Huh7 is another widely available cell line which was established in 1982 from a Japanese patient with a well differentiated hepatocellular carcinoma [24]. Huh7 cells maintain key hepatic functions and have been used in drug toxicity studies [25, 26], hepatic gene regulation [27], and hepatic disease models [28, 29]. Unfortunately, CYP450 gene expression and metabolism of Huh7 cells are also significantly lower than PHHs. DMSO treatment or 4-week of confluent culturing can improve their CYP450 expression. These methods stall cell division, upregulating CYP450 expression, and result in significant inducibility upon drug exposure [27, 30, 31]. Still, these methods are not common practice and have not yet been validated or compared to PHHs.

Recent advances in the understanding of cellular differentiation allow the generation of stem cell derived hepatocytes, as an alternative source of sustainable human hepatocytes. Characterization of human embryonic stem cell derived human hepatocytes (hESC-HHs) has shown high expression levels of mature hepatocyte proteins such as albumin and (alpha)1-antitrypsin, 75% and 64% that of PHHs, respectively [32]. However, they present undetectable levels of many CYP450 enzymes and a high expression of fetal CYP3A7, suggesting that this model needs further maturation [33]. Moreover, hESC-HHs are derived from the inner cell mass of a blastocyst, which raises ethical concerns that may limit their use. Induced pluripotent stem cell derived human hepatocytes (iPSC-HHs), on the other hand, are derived from somatic cells eliminating the need of embryonic tissue [34]. Moreover, they can be derived from patients with specific genetic variants and have shown a more complete hepatic phenotype with high expression levels of CYP450 enzymes, including CYP3A4, CYP2D6, CYP2C9, and CYP1A2, as they approach a mature hepatocytic stage [35–37]. Although detectable, their CYP450 expression does not match up to their PHH counterpart, and their CYP3A4 induction upon drug exposure and its correlation to PXR expression have not been fully characterized [38, 39].

**Fig 1:**
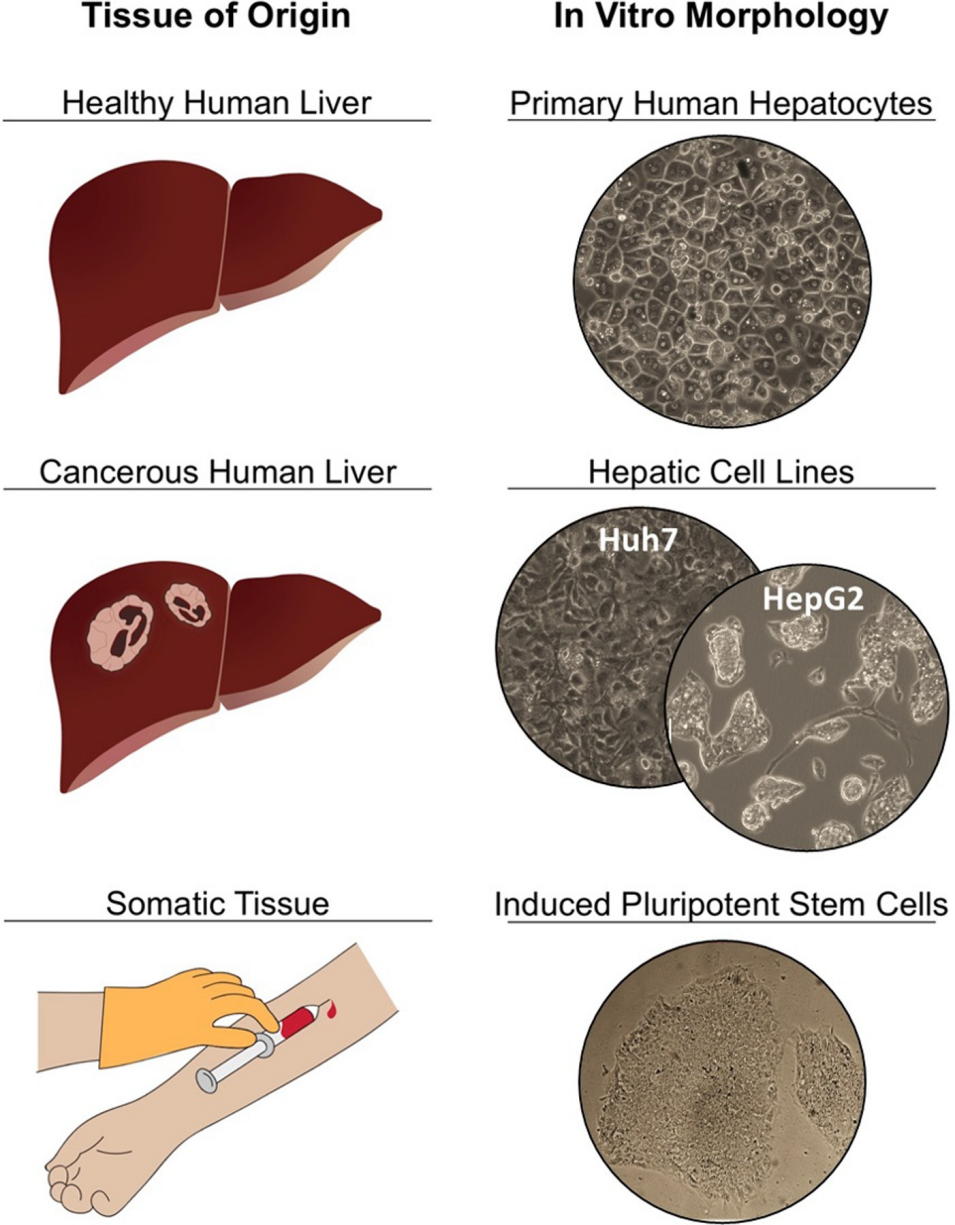
Illustration of different hepatic cell types used in this study. Four different *in vitro* platforms are investigated: primary human hepatocytes isolated from human livers rejected for transplantation; carcinoma derived hepatic cell lines Huh7 and HepG2; and somatic tissue derived induced pluripotent stem cells (iPSC), which are differentiated into hepatocytes.

Previously, various groups compared the CYP inducibility of HepG2s, PHHs, HepaRGs and Huh7s [21, 40–42] as well as different culture methods such as 2D plating versus 3D spheroid cultures [43]. In this study we analyzed and compared the CYP3A4 inducibility of freshly isolated and cryopreserved PHHs, and human hepatoma cell lines, HepG2 and Huh7, in addition to iPSC-HHs (Fig 1). The mRNA expression levels of CYP3A4 and PXR were determined after exposure to rifampicin, the most well-known and studied activator of human PXR. The effects of rifampicin on the transcription of both PXR and CYP3A4 were studied across these most common forms of available hepatocytic cellular platforms.

## Materials and methods

### Primary human hepatocyte (PHH) cultures

Fresh PHHs were purchased from the Cell Resource Core (CRC) at the Massachusetts General Hospital (MGH, Boston, MA, USA). Cryopreserved PHHs were purchased from the CRC and from Lonza (Walkersville, MD, USA). They were thawed according to the manufacturers’ instructions and plated onto collagen-I BioCoated 12 well plates (Corning, New York, NY, USA) at a density of 8.0 x 10^5^ cells per well in 0.5 mL of culture media at 95% confluency. For the first 24 hrs, prior to rifampicin treatment, the cells were maintained in Dulbecco’s Modified Eagle’s Medium (DMEM) (Life Technologies, Carlsbad, CA, USA) supplemented with 10% fetal bovine serum (FBS) (Sigma, St Louis, MO, USA), 0.5 U/mL insulin, 7 ng/mL glucagon, 20 ng/mL epidermal growth factor, 7.5 μg/mL hydrocortisone, 200 U/mL penicillin, 200 μg/mL streptomycin, and 50 μg/mL gentamicin. PHHs were incubated at 37°C and 10% CO_2_ throughout the experiments.

### Huh7 cultures

The human hepatoma cell line Huh7 was purchased from Creative Biolabs (Shirley, NY, USA) and thawed according to the manufacturer’s instructions. The cells were maintained in DMEM supplemented with 10% FBS, 200 U/mL penicillin and 200 μg/mL streptomycin and incubated at 37°C and 5% CO_2_. Huh7 cells were expanded in culture flasks, then detached using 0.5% trypsin-EDTA (Thermofisher Scientific, Waltham, MA, USA) and seeded in 12 well plates at a density of 2.0 x 10^5^ cells per well in 1 mL of culture media. After 24 hrs, the plates reached confluency and were further cultured for 0, 1, 2, 3 or 4 weeks, with daily media change, before rifampicin treatment.

### HepG2 cultures

The HepG2 hepatoma cell line was purchased from ATCC (Manassas, VA, USA) and thawed according to the manufacturer’s instructions. The cells were maintained in DMEM supplemented with 10% FBS, 200 U/mL penicillin, and 200 μg/mL streptomycin and incubated at 37°C and 5% CO_2_. HepG2 cells were expanded in culture flasks, then detached using 0.5% trypsin-EDTA and seeded in 12 well plates at a density of 2.0 x 10^5^ cells per well in 1 mL of culture media. After cell seeding, the cells were incubated for 24 hrs in their corresponding maintenance media prior to rifampicin treatment. For 4 week confluency experiments, the plates were further cultured for 4 weeks, with daily media change, before rifampicin treatment.

### iPSC culture and differentiation

All human iPSCs used in this study were previously generated [34, 37, 44–50]. For this study, six human iPSC lines including iHu71, iH34, iH14, iM9, iA3, and iH9 (either primary hepatocyte or fibroblast derived iPSCs) were used. This study was conducted in accordance with Johns Hopkins Institutional Stem Cell Research Oversight Committee regulations and following a protocol approved by the Johns Hopkins Institutional Review Board. The human iPSCs were cultured in feeder-free condition on Matrigel (BD), using mTeSR medium (Stem Cell Technologies, Vancouver, Canada) as previously described [34, 36, 44, 45]. The medium was replaced every day until the cells reached the desired confluency for passaging or differentiation. Once the human iPSCs reached approximately 50–70% confluency, endodermal commitment and hepatic differentiation was induced as previously described [36, 37, 48, 51]. Definitive endoderm (DE) stage cells were obtained after 3–5 days of iPSC culture in RPMI medium supplemented with B27 and 50–100 ng/mL activin A. Hepatic commitment was induced by culturing DE cells in RPMI medium supplemented with HGF and FGF4, 10 ng/ml each, for further 4–5 days to obtain hepatic progenitor (HP) stage cells. Hepatic maturation of HP cells to mature hepatocyte (MH) stage was stimulated by culturing HP cells for 10–15 days in William's medium E supplemented with 10 ng/mL HGF, FGF4, OSM and 0.1 μM dexamethasone. The iPSC derived hepatic progenitors and hepatocyte like cells were treated with rifampicin for 24 hrs as described below in the rifampicin treatment section.

### Rifampicin treatment

After the appropriate culturing period, the cells were treated with 0, 5, 10, or 20 μM rifampicin (Sigma-Aldrich, St Louis, MO, USA) in William’s medium E supplemented with 200 U/mL penicillin, 200 μg/mL streptomycin for 24 hrs. All groups were exposed to equal amounts of 0.2% DMSO. The experiments were performed at least in triplicate.

### RNA isolation and RT-PCR analysis

At the end of rifampicin treatment, the cells were lysed using Trizol (Thermofisher Scientific, Waltham, MA, USA). RNAs were isolated and purified using PureLink RNA Mini kit (Thermofisher Scientific, Waltham, MA, USA) following manufacturer’s instructions. A total of 800 ng of RNA were reverse transcribed using a cDNA synthesis kit (iScript, Bio-rad, Portland, ME, USA) according to the manufacturer’s instructions. The obtained cDNA was used for quantitative PCR analysis using Power SYBR Green PCR master mix kit (Life Technologies, Carlsbad, CA, USA). The reaction was performed in a ViiA 7 Real-time PCR system (Life Technologies, Carlsbad, CA, USA) according to the manufacturer’s instructions. The relative mRNA expression was quantified using the comparative Ct (ΔΔCt) method. PCR primers for PXR, CYP3A4 and the housekeeping genes B-actin, EIF1 are given in S1 Table.

RNAs from iPSC derivatives were extracted using TRIZOL (Invitrogen, CA, USA) based on manufacturer’s instructions. cDNA was obtained from mRNA using high capacity reverse transcription kit (Applied biosystems, CA, USA). Quantitative PCR analysis was performed to detect PXR and CYP3A4 expression using Taqman q-PCR probes including 18S (Hs03003631_g1), PXR (Hs01114267_m1), and CYP3A4 (Hs00604506_m1). The experiments were performed at least in triplicate.

### Statistical analysis

The data are presented as the mean ± standard error of the mean (SEM). For statistical analysis, GraphPad Prism software (Graphpad Software Inc., San Diego, CA) was used, where statistical significance of the results was assessed using one-way ANOVA. P-values less than 0.05 were considered statistically significant.

## Results

### Primary human hepatocytes

**Fig 2:**
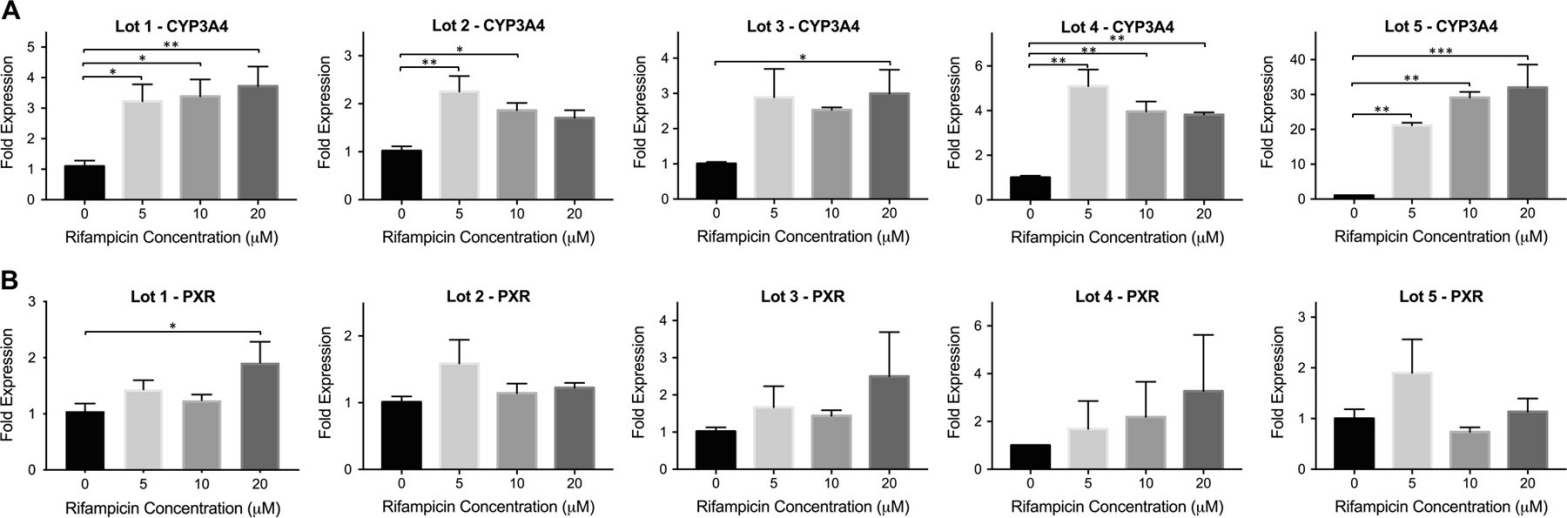
CYP3A4 and PXR expression in primary human hepatocytes (PHHs) upon rifampicin treatment. Cryopreserved PHHs from 5 different donors were incubated with increasing concentrations of rifampicin. Following 24 hrs incubation, the changes in the mRNA levels of **(A)** CYP3A4 and **(B)** PXR were quantified via quantitative real-time PCR. The data are presented as mean ± SEM. N≥3, * = p < 0.05, ** = p < 0.01, *** = p < 0.001.

We performed induction studies with five different cryopreserved primary hepatocyte lots from two different vendors as demonstrated in Fig 2. The donor information for each lot is given in Table 1. A great variability in CYP3A4 induction was observed among the different lots upon rifampicin treatment (Fig 2A). A maximum of 3.7, 2.2, 3.0, 5.1, and 32-fold CYP3A4 induction was observed for lots 1, 2, 3, 4 and 5, respectively. For lots 1, 4 and 5, statistically significant induction was achieved at all rifampicin concentrations (5, 10 and 20 μM). For lot 2, significant increase in CYP3A4 levels were observed in the 5 and 10 μM rifampicin treated groups, whereas for lot 3, only 20 μM rifampicin treated group showed significant induction. Significant PXR induction was only observed for lot 1, where 1.9-fold increase was achieved in the 20 μM rifampicin treated group (Fig 2B). For lots 3 and 4, PXR levels were increased by 2.5 and 3.3-fold, however these increases were not significant due to high variation among biological replicates.

**Table 1:** Donor information of primary human hepatocytes.

Donor	Sex	Age	Ethnicity	BMI	ALT	AST	T BILI	Alch	Drug	Fat%	COD
**1**	M	45	Caucasian	25.6	90	110	1.0	Daily	None	0%	Suicide
**2**	F	16	Caucasian	48.0	45	23	0.5	None	None	3–5%	Head Trauma
**3**	F	25	Caucasian	20.3	174	198	0.2	None	None	0%	Cardiac Arrest
**4**	F	47	Caucasian	46.7	59	34	-	Socially	None	0%	Anoxia
**5**	M	3	Caucasian	16.8	-	-	-	None	None	-	Anoxia

M: Male, F: Female, BMI: Body mass index, ALT: Alanine aminotransferase (U/L), AST: Aspartate aminotransferase (U/L), T BILI: Total bilirubin (mg/dL), Alch: Alcohol use frequency, COD: Cause of death.

Freshly isolated samples were also available for lots 1, 2 and 3 such that we could assess and compare their inducibility with cryopreserved samples (S1 Fig). As shown in S1A Fig, 36, 3.3 and 25-fold CYP3A4 induction was achieved in fresh lots 1, 2 and 3, respectively. Significant PXR induction was observed in freshly isolated lot 3, in the 5 μM rifampicin treated group. While CYP3A4 inducibility stayed the same for lot 2 among the freshly isolated and cryo-preserved cells, it decreased significantly for lots 1 and 3 following cryo-preservation (S1B Fig).

### Hepatic cell lines

**Fig 3:**
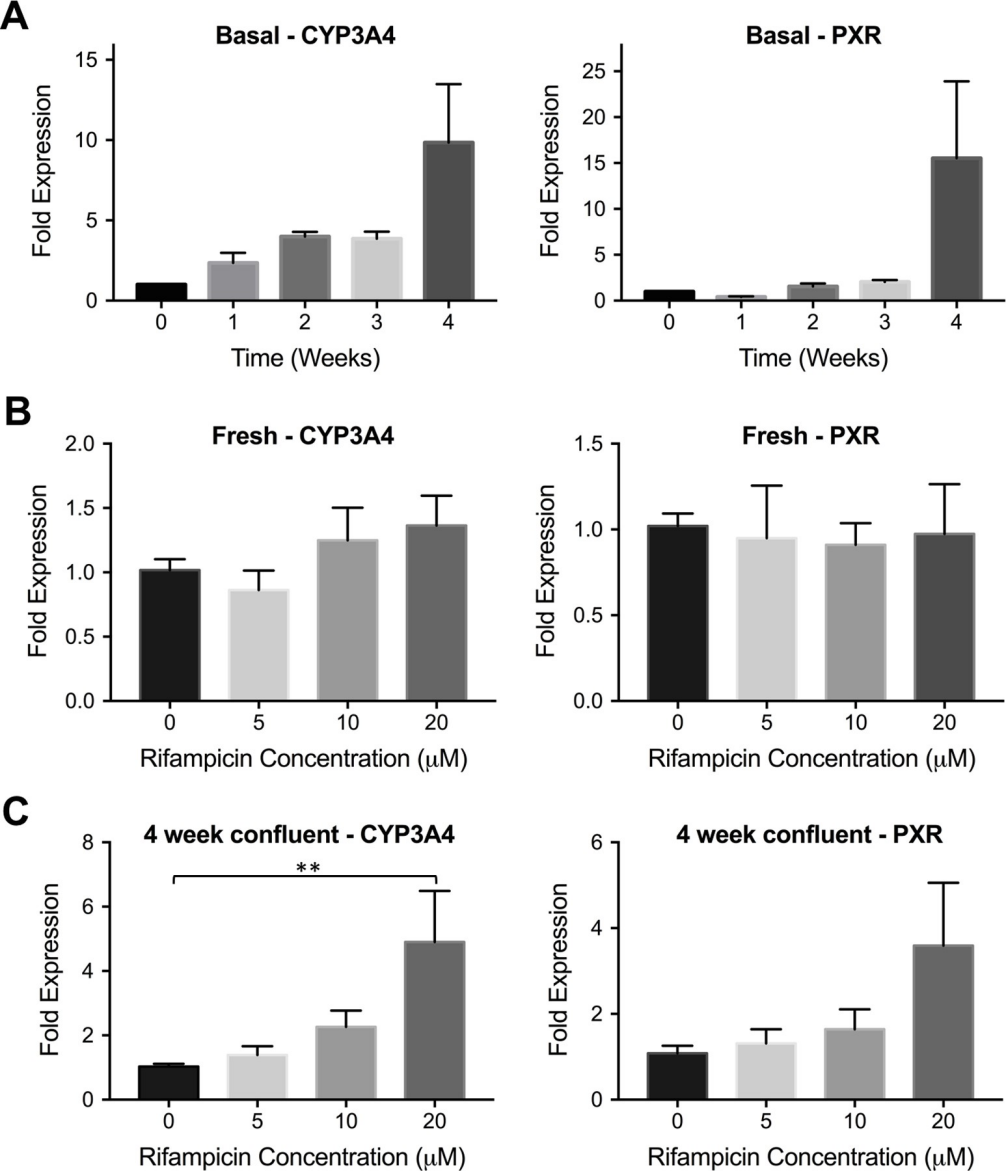
CYP3A4 and PXR expression in hepatic cell line Huh7. **(A)** Huh7 cells were cultured confluently for 4 weeks. The changes in basal CYP3A4 and PXR levels were quantified via quantitative real-time PCR. The data are presented as mean ± SEM, N≥6. **(B)** Freshly plated Huh7s were incubated with increasing rifampicin concentrations for 24 hrs. The relative mRNA levels of CYP3A4 and PXR compared to non-treated cells are shown. The data are presented as mean ± SEM, N = 8. **(C)** Huh7s were cultured confluently for 4 weeks. At the end of week 4, the cells were treated with rifampicin for 24 hrs. The mRNA levels of CYP3A4 and PXR were quantified. The data are presented as mean ± SEM. N≥9, ** = p < 0.01.

We performed rifampicin induction studies in two different cell lines: Huh7 and HepG2. A previous study showed that basal CYP3A4 levels were increased in confluently cultured Huh7 cultures [27, 31]. We cultured this cell line confluently for up to 4 weeks and analyzed the fold expression levels of both CYP3A4 and PXR at different time points (weeks 1, 2, 3 and 4) as shown in Fig 3A. At the end of 4 weeks, we found that CYP3A4 expression was upregulated by 9.8-fold whereas PXR levels were increased by 15-fold compared to freshly plated cells. We also performed induction studies via rifampicin treatment in both freshly plated and confluently cultured Huh7s. In fresh Huh7s, rifampicin did not result in a significant CYP3A4 or PXR induction whereas 4-week confluent culturing improved the inducibility of this cell line, resulting in 4.9 and 3.6-fold induction in CYP3A4 and PXR levels, respectively (Fig 3B and 3C).

**Fig 4:**
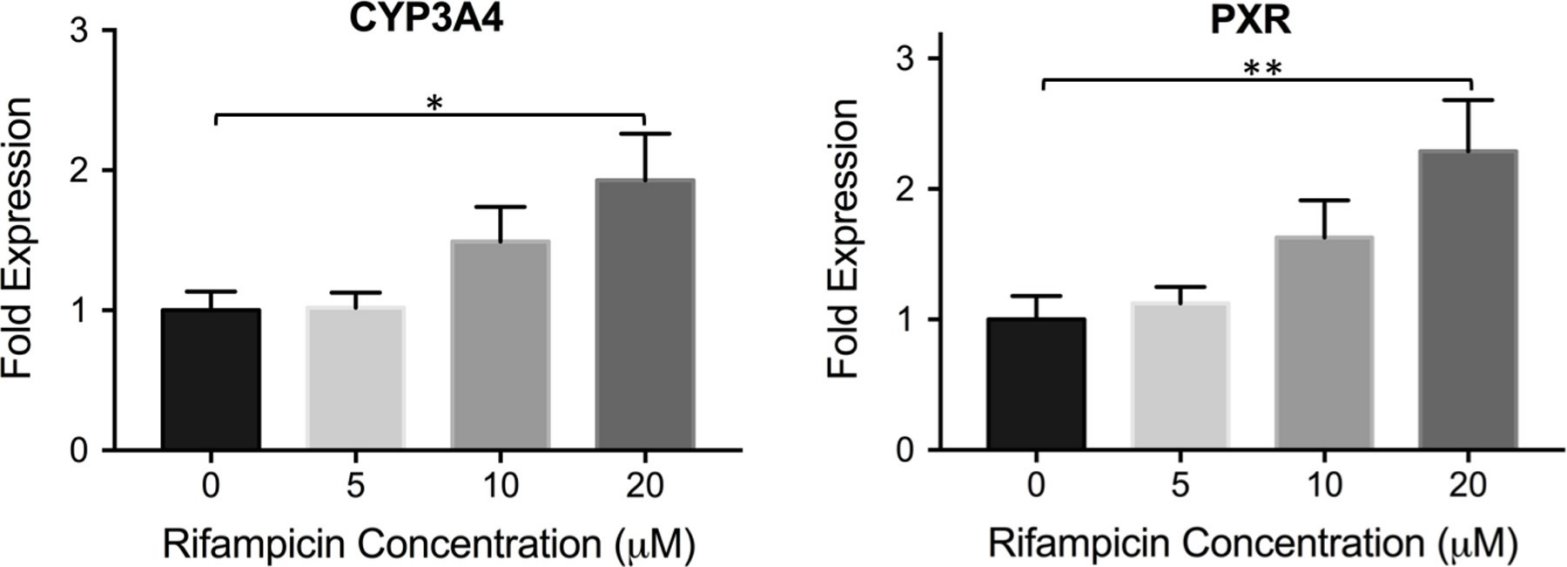
CYP3A4 and PXR expression in hepatic cell line HepG2 upon rifampicin treatment. HepG2 cells were treated with different rifampicin concentrations for 24 hrs. The fold change in expression levels of CYP3A4 and PXR are shown. The data are presented as mean ± SEM, N = 6, * = p < 0.05, ** = p < 0.01.

In HepG2 cultures, a gradual increase in the expression levels of both CYP3A4 and PXR was observed upon treatment with increasing rifampicin concentrations as shown in Fig 4. The maximum induction was achieved in the 20 μM rifampicin treated group, where 1.9 and 2.3-fold increases were observed in the expression levels of CYP3A4 and PXR, respectively. Upon achieving increased inducibility following 4-week confluent culturing in Huh7 cells, we applied the same approach to HepG2 cells. After culturing the cells confluently for 4 weeks, we looked at the induction levels following rifampicin treatment, as well as changes in basal CYP3A4 and PXR expression. As shown in S2A Fig, a slightly increased fold expression was observed for CYP3A4 in the 20 μM rifampicin treated group (2.1-fold) following confluent culturing, compared to freshly treated cells. No statistically significant changes in PXR levels were achieved following rifampicin treatment at 4-week confluency. In addition, the basal expression levels of both CYP3A4 and PXR were decreased upon 4-week confluent culturing (S2B Fig).

In addition, the basal expression levels of both CYP3A4 and PXR were higher in HepG2 cells compared to Huh7 cells cultured for 0–4 weeks as demonstrated in S3 Fig. Three out of five PHH batches (donors 1, 4 and 5) had higher CYP3A4 mRNA levels compared to HepG2s whereas HepG2s had the highest PXR expression compared to PHHs except for donor 5 (S3 Fig).

### Induced pluripotent stem cell (iPSC) derived human hepatocytes

**Fig 5:**
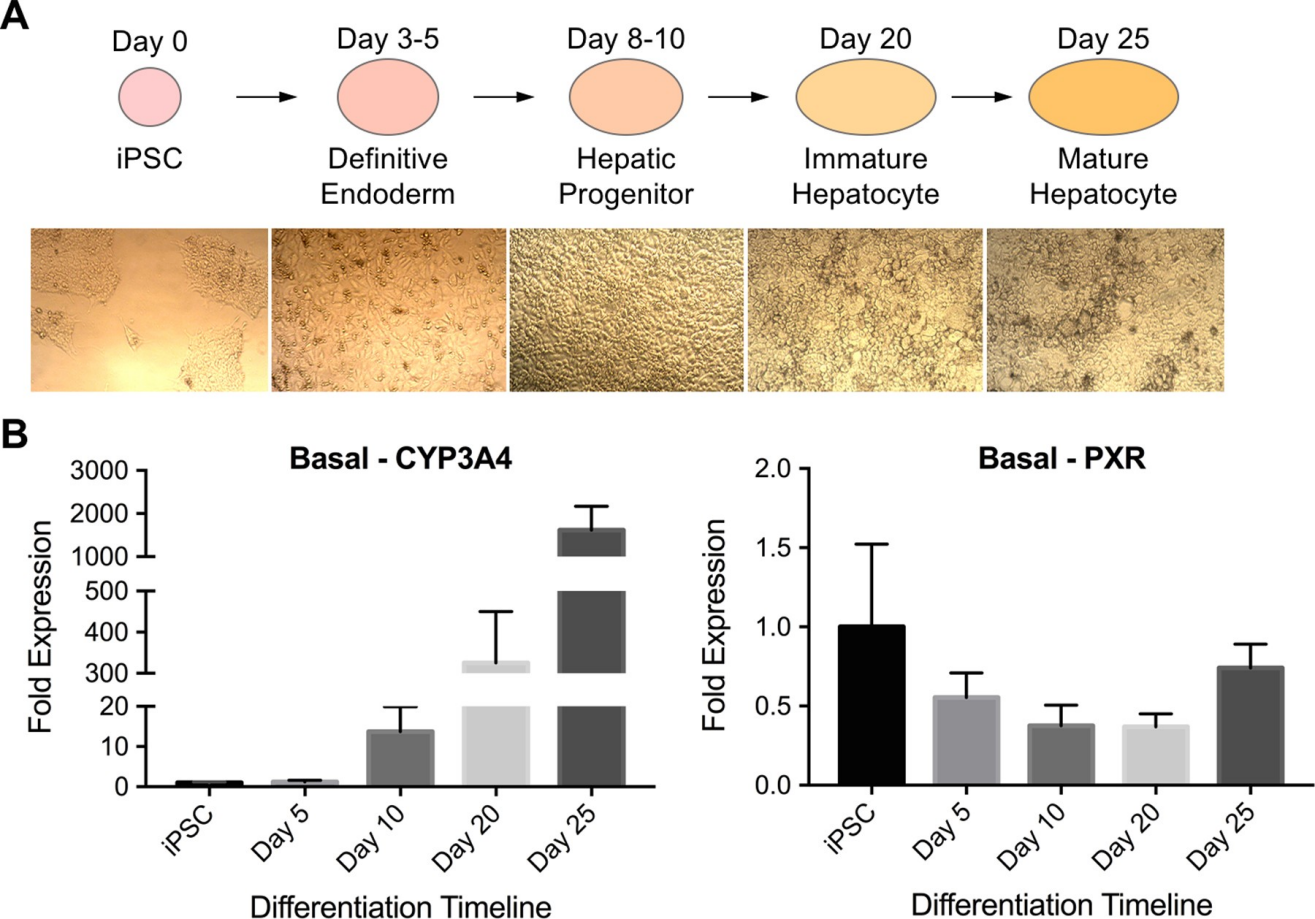
Basal CYP3A4 and PXR expression in differentiating human iPSCs. **(A)** Human induced pluripotent stem cells are differentiated into hepatocyte like cells via a 25-day long protocol. Representative pictures of cells at different differentiation stages are presented. **(B)** The basal expression levels of CYP3A4 and PXR were quantified at different differentiation stages and are normalized against undifferentiated iPSC cells. The data are presented as mean ± SEM, N = 6.

Pluripotent stem cells can be differentiated into many different cell types, including hepatocytes. For this study, we employed a 25-day long protocol, where pluripotent stem cells differentiate into definitive endoderm- (day 3–5), hepatic progenitor- (day 8–10), immature hepatocyte- (day 20) and finally into mature hepatocyte- like cells (day 25) (Fig 5A) [37, 48, 51, 52]. We looked at the basal expression levels of CYP3A4 and PXR at different stages of the differentiation (on days 5, 10, 20 and 25). A gradual increase in CYP3A4 expression was observed as the differentiation progressed, whereby over 1500-fold increase was achieved at the final differentiation stage (mature hepatocyte), compared to undifferentiated iPSCs. This high fold increase is most likely due to the very low initial expression of CYP3A4 in undifferentiated iPSCs. On the other hand, PXR expression did not show significant changes during the differentiation procedure (Fig 5B). We also performed albumin immunostaining on mature hepatocyte-like cells and showed that the cells are producing albumin and that the albumin production among the non-treated and 20 μM rifampicin treated groups was similar (S4 Fig).

**Fig 6:**
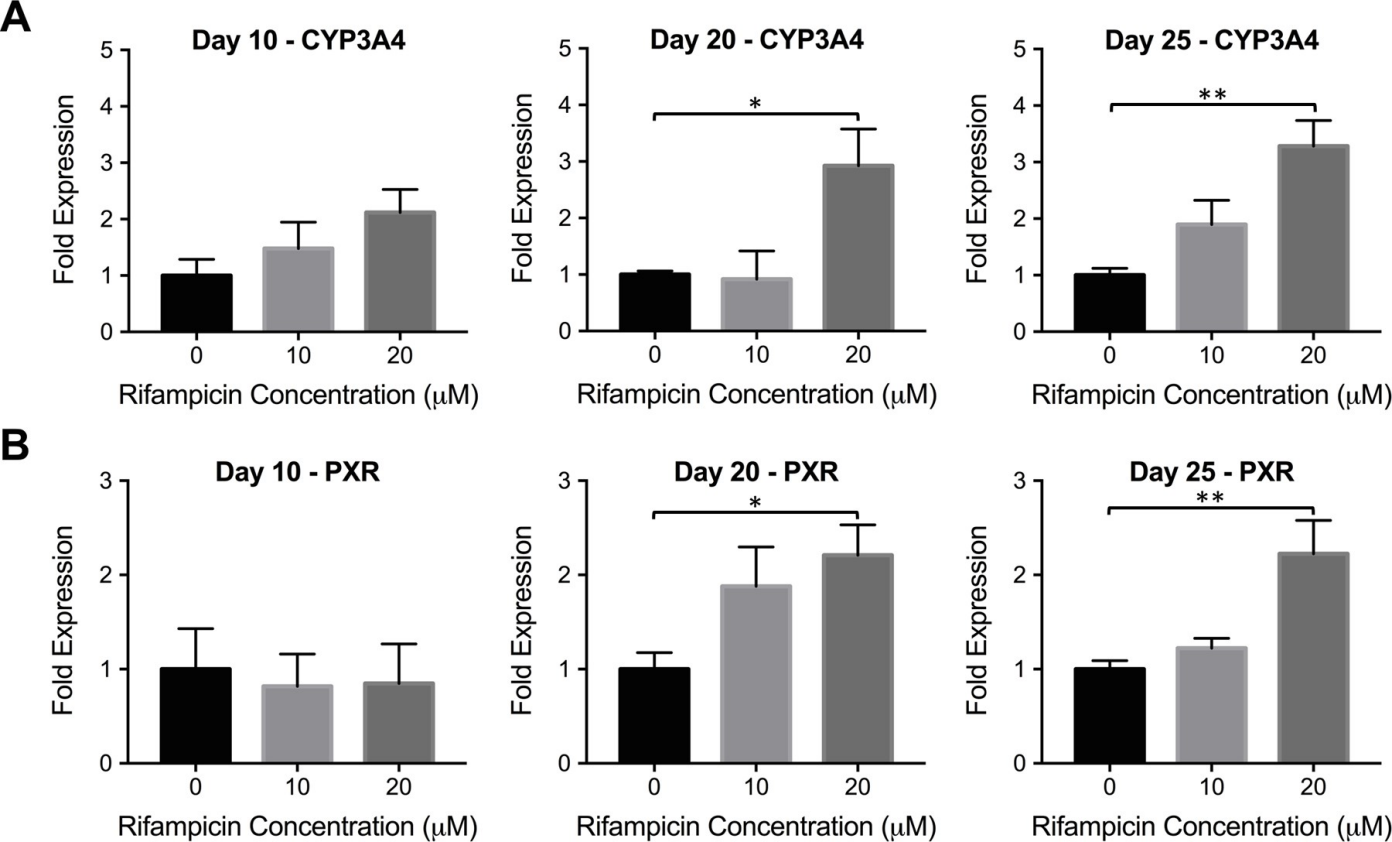
CYP3A4 and PXR induction in hepatic progenitors, immature and mature hepatocyte like cells differentiated from human iPSCs. Human iPSCs-derived hepatocytic cells were exposed to increasing rifampicin concentrations at different differentiation stages: day 10 (hepatic progenitor stage), day 20 (immature hepatocyte stage) and day 25 (mature hepatocyte stage). Following 24 hrs incubation, the changes in the mRNA levels of **(A)** CYP3A4 and **(B)** PXR were quantified via quantitative real-time PCR. The data are presented as mean ± SEM, N≥5, * = p < 0.05, ** = p < 0.01.

We treated differentiated cells from human iPSCs on days 10 (hepatic progenitor stage), 20 (immature hepatocyte stage) and 25 (mature hepatocyte stage) with rifampicin and investigated CYP3A4 and PXR expression levels, as shown in Fig 6. Statistically significant changes in CYP3A4 and PXR expression were observed only with immature and mature hepatocyte stage cells in the 20 μM rifampicin treated group. Compared to non-treated groups, 2.9 and 3.3-fold CYP3A4 induction was observed for immature and mature hepatocyte like cells, respectively (Fig 6A). Similarly, 2.2-fold PXR induction was observed for both immature and mature hepatocyte like cells (Fig 6B).

## Discussion

Cytochrome P450 (CYP450) enzymes are responsible for the phase I drug metabolism in the liver. Among these enzymes, CYP3A4 is involved in the metabolization of most drugs and its expression is regulated mainly by the nuclear receptor pregnane X receptor (PXR) among others. Different *in vitro* platforms are employed to study drug inducibility and drug-drug interactions, including primary hepatocytes, hepatic cell lines as well as stem cell derived hepatocytes. In this work, we present a comprehensive study of CYP3A4 induction in primary human hepatocytes, hepatic cell lines HepG2 and Huh7, and iPSC derived human hepatocytes (Fig 1). In addition, we also investigated the changes in PXR expression levels in all of these platforms upon induction, which has not been studied in as much detail before.

We tested the inducibility of primary human hepatocytes acquired from two different companies with a donor age range of 3–47 (Table 1). The results showed great variability among the five different lots as demonstrated in Fig 2 and no correlation was observed between the donor ages or body mass index (BMI) levels and CYP3A4 inducibility. Lot 5 achieved high CYP3A4 induction levels (32-fold at 20 μM rifampicin), whereas other donors were inducible by up to 5-fold only. There was no clear correlation between the changes in expression levels of CYP3A4 and PXR; and significant increase in PXR expression was achieved for only lot 1. We also looked at the induction levels in freshly isolated cells of three different donors (lots 1, 2 and 3) as shown in S1 Fig, and found that the cells lost their CYP3A4 inducibility by up to 90% after the cryo-preservation and thawing process. A decrease in PXR inducibility was also observed upon cryo-preservation, and interestingly, statistically significant increase in PXR levels was observed for freshly-plated lot 3 hepatocytes only (S1A Fig).

In literature, there are contradictory findings regarding the effect of cryopreservation on CYP expression and inducibility. A study conducted with PHHs showed a slightly decreased expression of CYP3A4 in cryo-preserved cells upon rifampicin treatment [53], whereas another study by Li *et al*. showed increased CYP3A4 activity in PHHs following cryo-preservation [54]. Another study conducted with primary rat hepatocytes also showed similar CYP P450 induction in freshly isolated and cryo-preserved cells [55]. Our findings agreed with the previous literature showing that cryopreservation can have a negative impact on CYP450 inducibility [56]. The heterogeneity of CYP3A4 induction among the different PHH lots combined with the loss in inducibility upon cryo-preservation suggest that alternative platforms, such as hepatic cell lines, can provide more reproducible and reliable results.

The CYP3A4 metabolism was impaired in the hepatic cell line Huh7, which did not show any inducibility upon rifampicin treatment when used directly after plating (Fig 3B). Previously, it has been shown that confluent culturing of this cell line resulted in increased basal expression levels of CYP3A4 via restorement of PXR activation [27, 31]. We cultured Huh7s confluently for 4 weeks and observed an increase in CYP3A4 (up to 9.8-fold) and PXR (up to 15-fold) basal expression levels (Fig 3A). Following this observation, we performed induction studies in 4-week confluent Huh7s. A gradual increase and a correlation in expression levels of CYP3A4 and PXR were observed (Fig 3C); and the highest CYP3A4 induction was achieved in the 20 μM rifampicin treated group with 4.9-fold increase in mRNA levels.

The other hepatic cell line HepG2 is one of the most widely used cell lines in drug induction studies. In contrast with primary hepatocytes, we observed a correlation between the changes in expression levels of CYP3A4 and PXR, where both proteins demonstrated a gradual increase in mRNA levels upon treatment with increasing rifampicin concentrations (Fig 4). A 1.9-fold CYP3A4 induction was achieved upon 20 μM rifampicin treatment, which was lower than what was observed with primary cells (Fig 2), in agreement with studies done by others where HepG2s were shown to respond weakly to drug treatment compared to PHHs or other cell lines such as HepaRGs [40, 42]. Following the promising results achieved in 4-week confluently cultured Huh7 cells, we cultured HepG2 cells for 4 weeks as well to investigate the effect of confluent culturing on this hepatic cell line. In contrast to Huh7s, we did not observe a significant improvement in CYP3A4 or PXR inducibility in confluently cultured cells (S2A Fig). In addition, the basal expression levels of both genes were downregulated in 4-week cultures, compared to freshly plated cells (S2B Fig), in contradiction to one study, where the authors looked at differential gene expression of CYP3A4 and PXR on culture days 3 and 21; and showed that CYP3A4 basal expression was higher in cells cultured for 21 days, whereas a significant increase in basal PXR levels was not observed [57].

Compared to both freshly plated and confluently cultured HepG2s, confluently cultured Huh7s performed better and compared closer to PHHs. Others also showed that Huh7 cells exhibited higher CYP3A4 activity compared to HepG2s [41]. Unlike the primary hepatocytes, both cell lines exhibited a matching increase in PXR expression levels upon drug treatment. Even though Huh7 cells at 4 weeks demonstrated a higher inducibility compared to HepG2s, they had the lowest basal CYP3A4 and PXR expressions compared to freshly plated HepG2s and PHH lots (S3 Fig). It is also important to note that HepG2 cells had comparable basal expression levels of both CYP3A4 and PXR as PHHs, which may imply that the basal CYP mechanism in HepG2s could be similar to those of PHHs unlike their ability to respond to drug induction.

The last platform we used in this study was iPSC derived human hepatocytes. Human induced pluripotent stem cell (iPSC) technology enables efficient preclinical drug studies using more disease-specific, human-relevant, and personalized cells/tissues. The iPSC-derived various cell types including iPSC-hepatocytes allow prediction of human responses with regards to both drug efficacy and toxicity. Patient-specific iPSCs have been used for *in vitro* modeling of various diseases and for drug screening and discovery [36, 47, 48, 50–52, 58, 59]. In addition to prediction of efficacy using patient-iPSC based disease models, iPSC-derived hepatocytes could address hepatotoxicity, a major barrier to drug approval. Unlike primary hepatocytes, iPSCs are renewable and amenable to gene editing [36, 60, 61]. These features are important for reliable drug testing and allow us to predict drug toxicities and metabolism that are associated with genetic polymorphism, which cannot be achieved using conventional tools. However, iPSC-derivatives cannot recapitulate all functions and complexity of human tissues in *in vivo* microenvironment. Together with continued improvements in iPSC technology, the potential of iPSC-hepatocytes in drug development is promising.

In this study, stem cells were differentiated into hepatocyte like cells accompanied by four differentiation stages: definitive endoderm (day 5), hepatic progenitor (day 10), immature hepatocyte (day 20) and mature hepatocyte (day 25) (Fig 5A) [36, 37, 48, 51, 52]. We quantified the basal CYP3A4 and PXR expression levels at each differentiation stage as shown in Fig 5. Interestingly, PXR levels did not change during the differentiation timeline whereas CYP3A4 levels increased by greater than 1500-fold at the final mature hepatocyte stage. This may imply that rather than an increase in the number of PXR molecules, an improvement in PXR activation mechanism took place as the stem cells progressed towards mature hepatocytes. We have also confirmed albumin production in day 25 hepatocytes, indicating the maturation status of the differentiated iPSC-derived cells as shown in S4 Fig.

We also studied inducibility in hepatic progenitor-, immature- and mature hepatocyte- stage cells (Fig 6). Hepatic progenitors did not demonstrate inducibility whereas significant CYP and PXR induction was observed in immature and mature hepatocyte stage cells. At the final differentiation stage, the CYP3A4 inducibility (by 3.3-fold) was comparable to some of the primary hepatocyte lots and to confluently cultured Huh7s. Similar to what was observed in hepatic cell lines, the changes in CYP3A4 expression were in correlation with the changes in PXR expression in both immature and mature hepatocyte stage cells differentiated from human iPSCs.

## Conclusion

It is vital to find an inducible and reproducible *in vitro* platform for drug toxicology and DDI studies. Aligned with these efforts, PHHs became the gold standard. However, they exhibit considerable variability, are expensive, and their CYP450 metabolism and inducibility are negatively affected by cryopreservation. As such, alternative sources have been utilized including hepatic cell lines and human stem cell derived hepatocytes. In this study, we provide a detailed comparison of the CYP3A4 and PXR induction in human PHHs, human hepatic cell lines HepG2 and Huh7, and human iPSC-derived hepatocytes. Our results showed that different levels of CYP3A4 induction can be achieved upon rifampicin treatment in all *in vitro* platforms. Considering the observed heterogeneity among the PHH lots, in addition to issues related with cost and availability, hepatic cell lines can be a viable alternative for PHHs, providing more reproducible results with less variation. After a maturation period (e.g. confluent culturing for Huh7s), similar CYP3A4 induction levels can be achieved in cell lines, as well as iPSC derived hepatocyte like cells, compared to PHHs. In addition, both iPSC derived hepatocytes and hepatic cell lines exhibited more reproducible PXR induction than PHHs. This can be attributed to maturation of the xenobiotic sensory system under the influence of PXR activation in cell lines or diminished xenobiotic response in cryopreserved PHHs. Further investigations on cells lines are needed to elucidate their full xenobiotic response capability, before their routine utilization for drug development studies.

## Supporting information

S1 Dataset (DOCX)

S1 Fig The CYP3A4 and PXR levels upon rifampicin treatment in fresh and cryo-preserved primary human hepatocytes. **(A)** Freshly isolated PHHs were exposed to increasing rifampicin concentrations. CYP3A4 and PXR levels were quantified via quantitative real-time PCR and plotted as fold expression normalized to the non-treated group. **(B)** Same experiment was performed with cryopreserved cells from the same lots. The data are presented as mean ± SEM, N = 3.
(DOCX)

S2 Fig CYP3A4 and PXR expression in hepatic cell line HepG2 following confluent culturing. HepG2 cells were cultured confluently for 4 weeks. **(A)** At the end of week 4, the cells were treated with rifampicin for 24 hrs. The mRNA levels of CYP3A4 and PXR were quantified. The data are presented as mean ± SEM. N = 6, * = p < 0.05. **(B)** The changes in basal CYP3A4 and PXR levels were quantified via quantitative real-time PCR. The data are presented as mean ± SEM, N = 6, ** = p < 0.01.
(DOCX)

S3 Fig Basal CYP3A4 and PXR levels in PHHs and cell lines. The basal CYP3A4 and PXR levels were quantified via quantitative real-time PCR in non-treated cells and were plotted as fold expression normalized to freshly cultured Huh7s.
(DOCX)

S4 Fig Albumin staining of human iPSC-derived mature hepatocytes. Human iPSC-derived hepatocyte-like cells are shown to be expressing a functional hepatocyte marker, albumin, at mature stage (day 25) after hepatic differentiation. The fluorescence intensity of albumin proteins was not significantly different among the untreated group and 20 μM rifampicin treated cells.
(DOCX)

S1 Table Sequences of qPCR primers. (DOCX)
