# Supplementary figures and images for: A comparison of hepato-cellular in vitro platforms to study CYP3A4 induction
Source: PLoS One. 2020 Feb 27;15(2):e0229106. doi: 10.1371/journal.pone.0229106 (PMC7046200; doi:10.1371/journal.pone.0229106)

**Data Set**

**
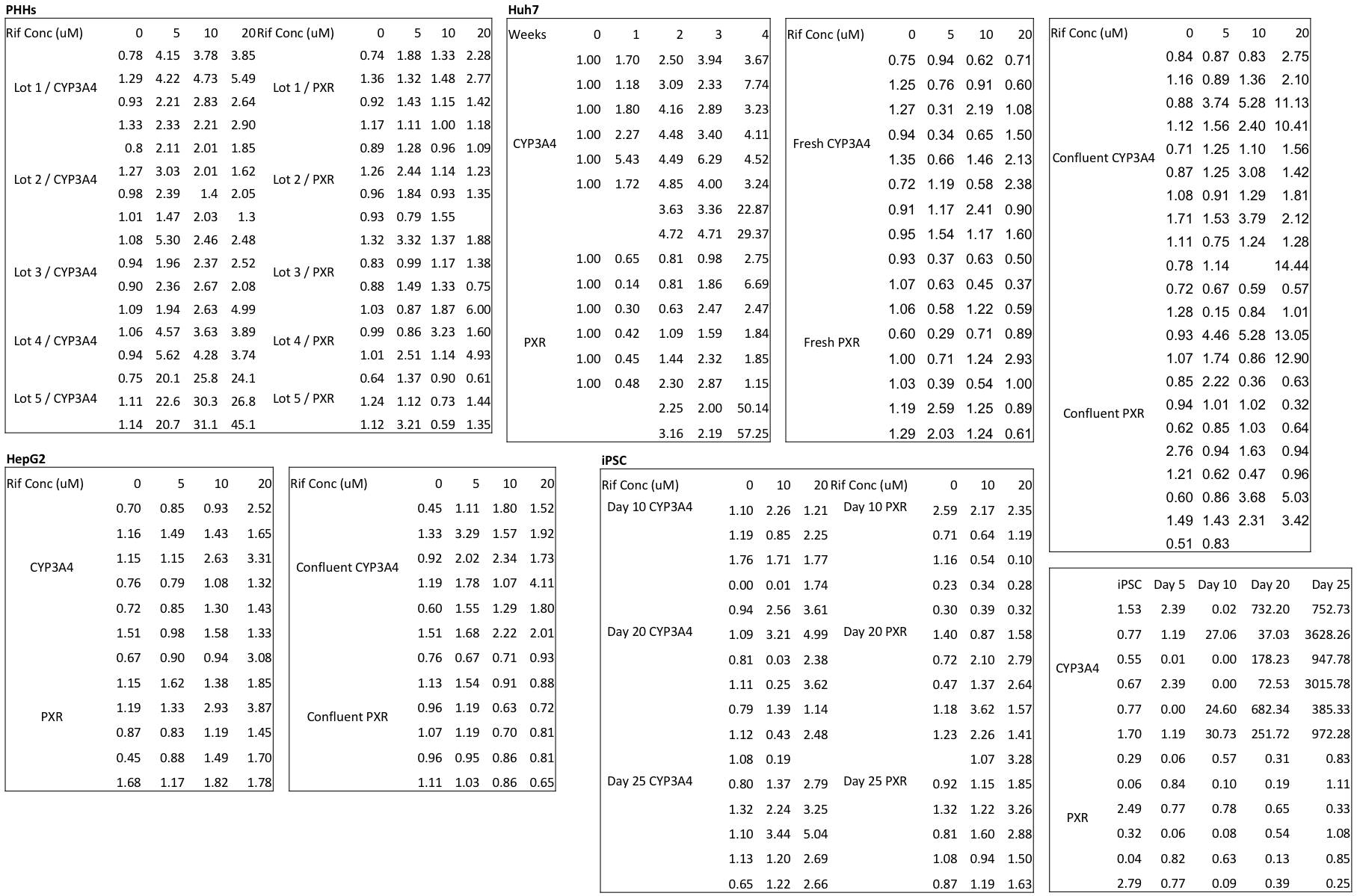
**

Supplement: S1 Dataset — (DOCX) [file pone.0229106.s001.docx]
