# Supplementary material for: A comparison of hepato-cellular in vitro platforms to study CYP3A4 induction
Source: PLoS One. 2020 Feb 27;15(2):e0229106. doi: 10.1371/journal.pone.0229106 (PMC7046200; doi:10.1371/journal.pone.0229106)
Supplement: S1 Fig — (A) Freshly isolated PHHs were exposed to increasing rifampicin concentrations. CYP3A4 and PXR levels were quantified via quantitative real-time PCR and plotted as fold expression normalized to the non-treated group. (B) Same experiment was performed with cryopreserved cells from the same lots. The data are presented as mean ± SEM, N = 3. (DOCX) [file pone.0229106.s002.docx]

**
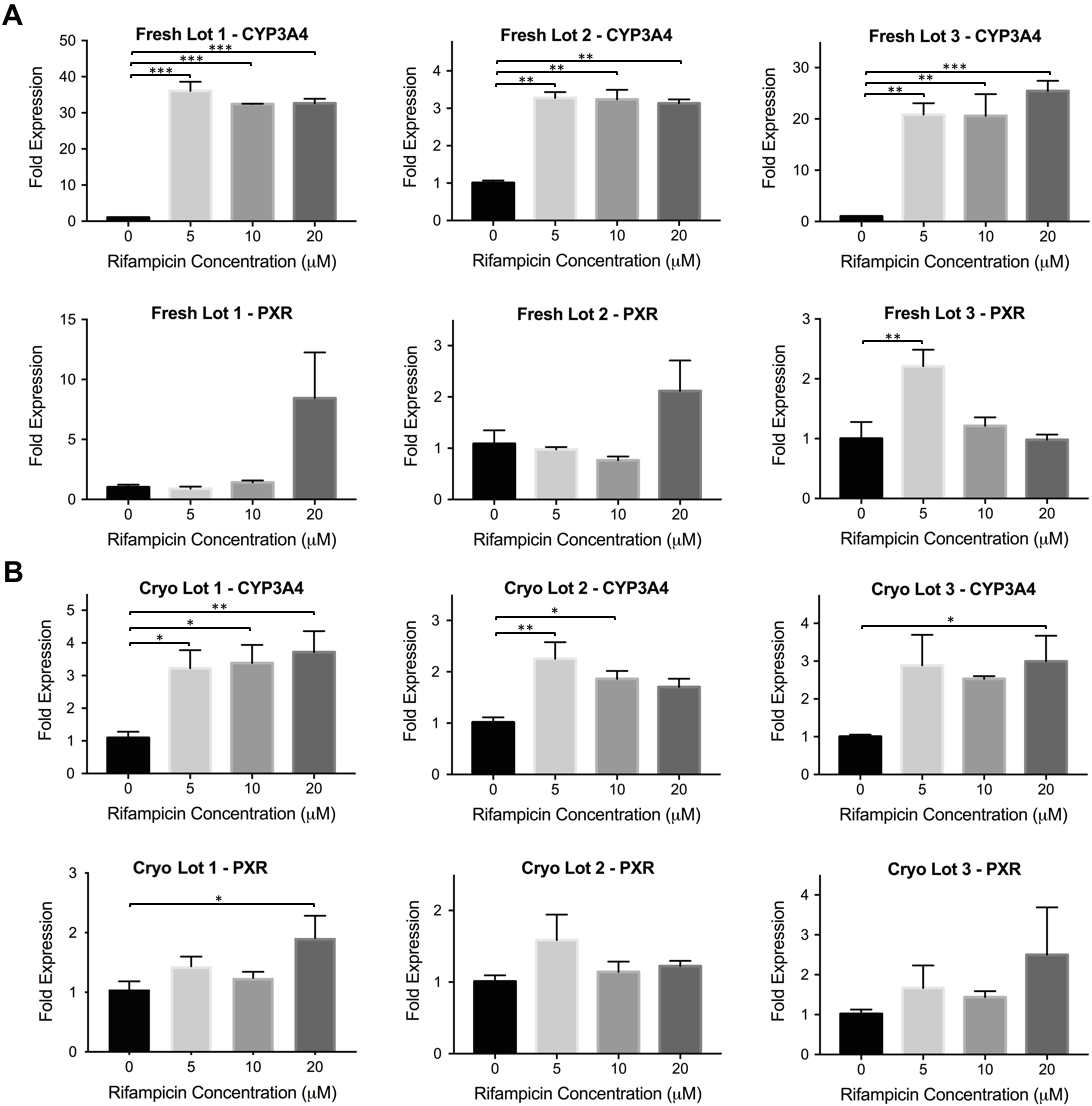
**

**S1 Fig. The CYP3A4 and PXR levels upon rifampicin treatment in fresh and cryo-preserved primary human hepatocytes. (A)** Freshly isolated PHHs were exposed to increasing rifampicin concentrations. CYP3A4 and PXR levels were quantified via quantitative real-time PCR and plotted as fold expression normalized to the non-treated group. **(B)** Same experiment was performed with cryopreserved cells from the same lots. The data are presented as mean ± SEM, N≥3, * = p < 0.05, ** = p < 0.01, *** = p < 0.001.
