# Supplementary material for: A comparison of hepato-cellular in vitro platforms to study CYP3A4 induction
Source: PLoS One. 2020 Feb 27;15(2):e0229106. doi: 10.1371/journal.pone.0229106 (PMC7046200; doi:10.1371/journal.pone.0229106)
Supplement: S2 Fig — HepG2 cells were cultured confluently for 4 weeks. (A) At the end of week 4, the cells were treated with rifampicin for 24 hrs. The mRNA levels of CYP3A4 and PXR were quantified. The data are presented as mean ± SEM. N = 6, * = p < 0.05. (B) The changes in basal CYP3A4 and PXR levels were quantified via quantitative real-time PCR. The data are presented as mean ± SEM, N = 6, ** = p < 0.01. (DOCX) [file pone.0229106.s003.docx]

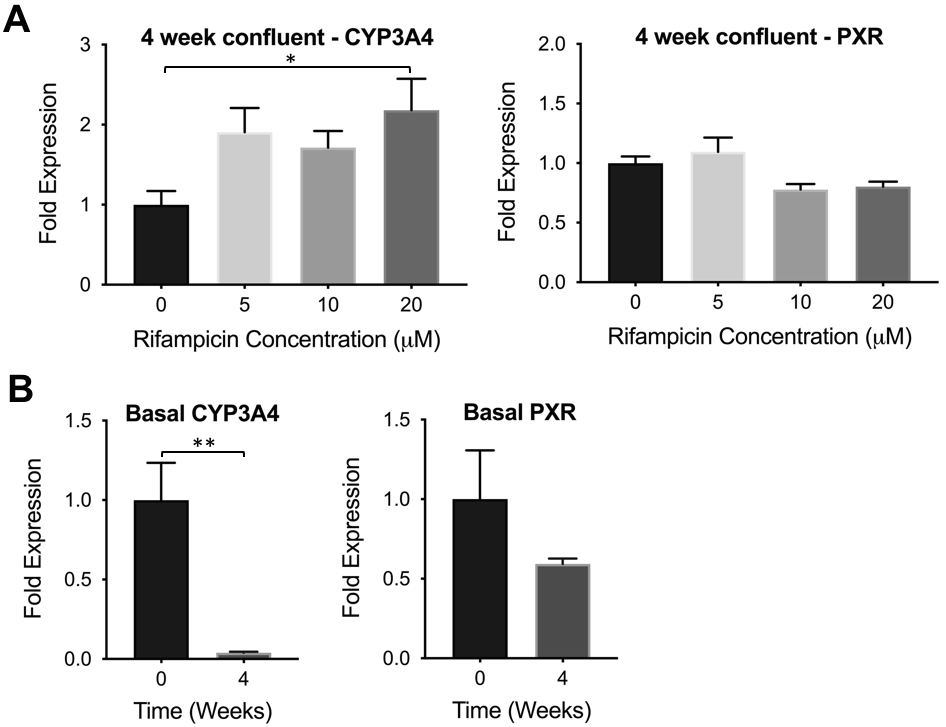


**S2 Fig. CYP3A4 and PXR expression in hepatic cell line HepG2 following confluent culturing.** HepG2 cells were cultured confluently for 4 weeks. **(A)** At the end of week 4, the cells were treated with rifampicin for 24 hrs. The mRNA levels of CYP3A4 and PXR were quantified. The data are presented as mean ± SEM. N=6, * = p < 0.05. **(B)** The changes in basal CYP3A4 and PXR levels were quantified via quantitative real-time PCR. The data are presented as mean ± SEM, N=6, ** = p < 0.01.
