# Supplementary material for: A comparison of hepato-cellular in vitro platforms to study CYP3A4 induction
Source: PLoS One. 2020 Feb 27;15(2):e0229106. doi: 10.1371/journal.pone.0229106 (PMC7046200; doi:10.1371/journal.pone.0229106)
Supplement: S3 Fig — The basal CYP3A4 and PXR levels were quantified via quantitative real-time PCR in non-treated cells and were plotted as fold expression normalized to freshly cultured Huh7s. (DOCX) [file pone.0229106.s004.docx]

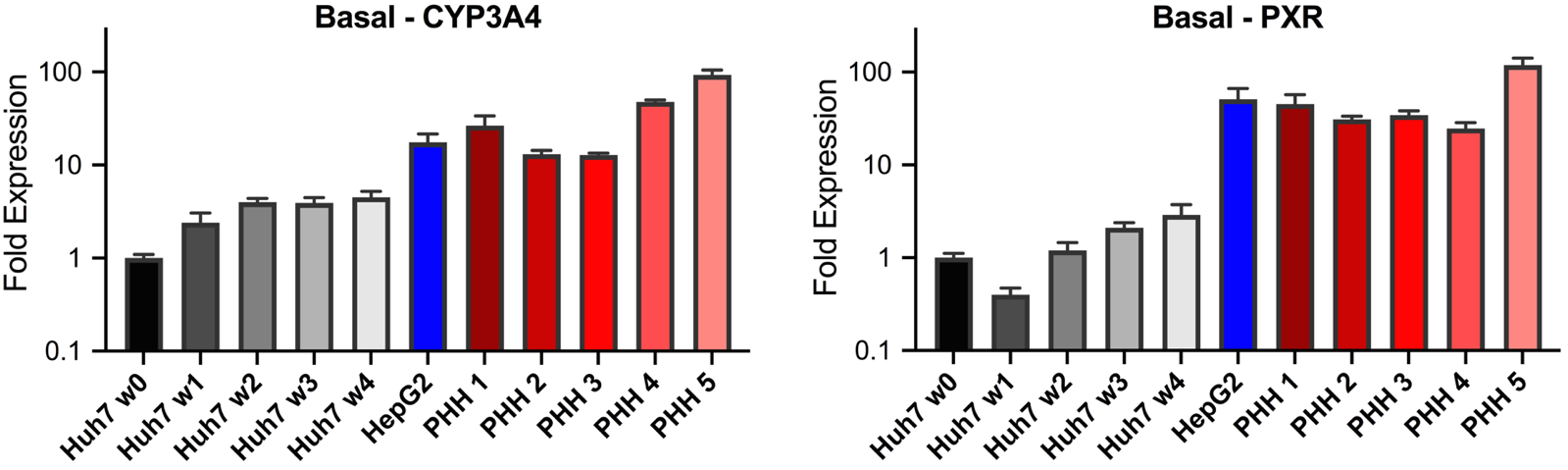
**S3 Fig. Basal CYP3A4 and PXR levels in PHHs and cell lines.** The basal CYP3A4 and PXR levels were quantified via quantitative real-time PCR in non-treated cells and were plotted as fold expression normalized to freshly cultured Huh7s. The data are presented as mean ± SEM, N≥3.
