# Supplementary material for: A comparison of hepato-cellular in vitro platforms to study CYP3A4 induction
Source: PLoS One. 2020 Feb 27;15(2):e0229106. doi: 10.1371/journal.pone.0229106 (PMC7046200; doi:10.1371/journal.pone.0229106)
Supplement: S4 Fig — Human iPSC-derived hepatocyte-like cells are shown to be expressing a functional hepatocyte marker, albumin, at mature stage (day 25) after hepatic differentiation. The fluorescence intensity of albumin proteins was not significantly different among the untreated group and 20 μM rifampicin treated cells. (DOCX) [file pone.0229106.s005.docx]

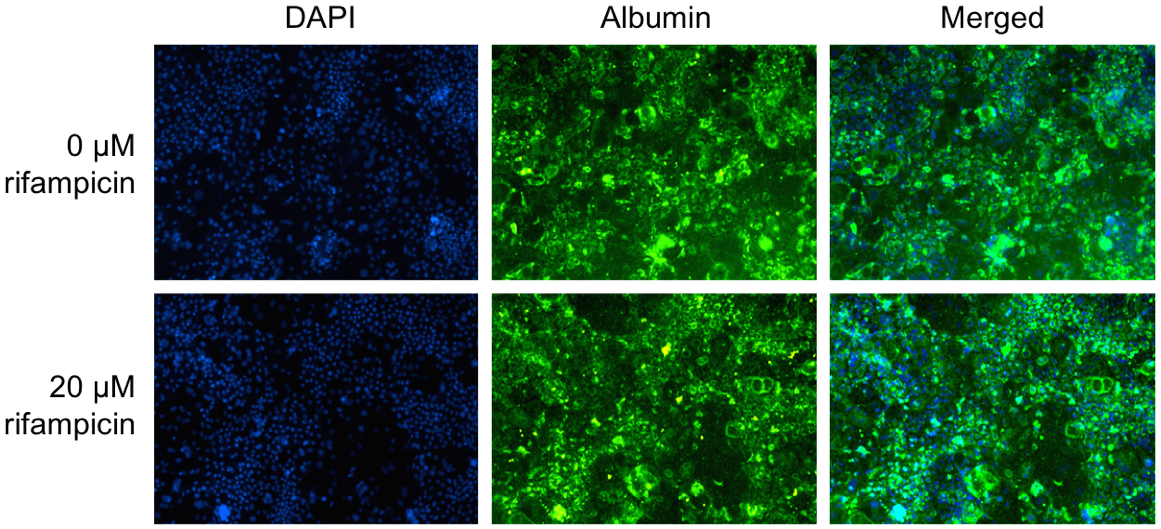


**S4 Fig. Albumin staining of human iPSC-derived mature hepatocytes.** Human iPSC-derived hepatocyte-like cells are shown to be expressing a functional hepatocyte marker, albumin, at mature stage (day 25) after hepatic differentiation. The fluorescence intensity of albumin proteins was not significantly different among the untreated group and 20 μM rifampicin treated cells.
