# Supplementary material for: A comparison of hepato-cellular in vitro platforms to study CYP3A4 induction
Source: PLoS One. 2020 Feb 27;15(2):e0229106. doi: 10.1371/journal.pone.0229106 (PMC7046200; doi:10.1371/journal.pone.0229106)
Supplement: S1 Table — (DOCX) [file pone.0229106.s006.docx]

**S1 Table.** Sequences of qPCR Primers.

| **Target** | **Forward sequence** | **Reverse sequence** |
| --- | --- | --- |
| **hPXR** | 5’ AGCTTTCCCACCCTCTTTGG 3’ | 5’ CTGAACAGTGTGCTCTGGGG 3’ |
| **CYP3A4** | 5’ AATCACTGTTGGCGTGGGG 3’ | 5’ AATGGGCAAAGTCACAGTGGA 3’ |
| **B-actin** | 5’ CCTCGCCTTTGCCGATCC 3’ | 5’ GCGCGGCGATATCATCATCC3’ |
| **EIF1** | 5’ AACCATTTGGGGTCCGCTTT 3’ | 5’ GCGCCTATTGCTTGACCTCT 3’ |
